# Supplementary material for: Are carbon emissions trading and green financial instruments synergistic? -Comprehensive quantitative research based on content analysis
Source: PLoS One. 2024 Mar 7;19(3):e0298601. doi: 10.1371/journal.pone.0298601 (PMC10919601; doi:10.1371/journal.pone.0298601)
Supplement: S1 Data — (ZIP) [file pone.0298601.s001.zip › Data/地级市Z_F工作报告.zip!/╣π╢1⁄2/╔╟═╖╩╨/└┤╫╘╣1⁄2╓┌║┼í╛┬φ┐╦╩2╛▌═°í┐_2003.docx]

各位代表：

我代表市人民政府向大会作政府工作报告，请予审议，并请各位政协委员和其他列席人员提出意见。过去五年工作回顾本届政府于1998年8月就职。五年来，市政府在市委的正确领导下，在市人大及其常委会和市政协的监督支持下，面对经济和社会发展过程中出现的各种困难和矛盾，团结带领全市人民，以经济建设为中心，在发展中克服困难，在前进中化解矛盾，国民经济和社会各项事业有了新发展。去年，完成国内生产总值483．29亿元，人均国内生产总值10271元，比1997年增长35．4％和18％，年均增长6．2％和3．4％；地方财政收入42．31亿元，比1997年增长45．8％，年均增长7．8％。

　　——经济结构调整取得成效。去年，全市工业总产值839．02亿元，比1997年增长49．4％，年均增长8．4％；工业增加值占国内生产总值的比重从1997年的40．23％提高到43．98％。扶持工业发展的力度加大，工业用电、用地价格大幅度下降。对重点工业项目组织现场办公，帮助企业解决生产经营中的问题，在全社会形成扶持工业的氛围。超声电子、纺织服装、化工塑料、机械装备、食品加工、印刷包装等传统产业得到改造提升，电子信息、生物工程和新材料等新兴产业有新发展。实施名牌带动战略取得成效，五年创立中国驰名商标2件，广东省著名商标33件，一批产品成为国家免检产品和省级名牌产品。澄海获得“中国玩具礼品城”称号。

　　特色农业、城郊农业和高效农业进一步发展。去年，全市农业总产值88．03亿元，比1997年增长19．6％，年均增长3．6％。果蔬、畜牧、水产业成为农村经济新增长点。建成“三高”农业示范基地30个，34家龙头企业带动12．79万农户增加收入。新制订地方性农业标准40项，位于全省前列。取得“广东省无公害农产品生产基地”认证22个。完成省下达的森林资源保护和发展目标。渔业基础设施进一步完善，海洋经济稳步发展。江海堤围工程建设、中小型水库除险加固、韩江下游五大出海口闸重建和练江片区内涝整治工程建设稳步推进，防灾抗灾能力进一步增强。

　　第三产业持续发展。去年全市第三产业增加值207．16亿元，比1997年增长31．7％，年均增长5．7％。现代物流业发展规划通过专家评审，会展经济活跃，新型流通业态逐步形成，一批国内外大型商贸零售企业相继落户汕头。去年，全社会消费品零售总额240．53亿元，比1997年增长56．9％，年均增长9．4％。金融、保险、证券业稳步发展。去年底，金融机构存款余额638．2亿元，比去年初增长16．4％；贷款余额419．2亿元，比去年初增长8．8％。房地产、汽车、文化、教育等新的消费热点形成。五年商品房销售面积653．52万平方米，销售额145．06亿元。旅游业迅速发展，进入“中国优秀旅游城市”行列。去年成功举办第一届汕头市旅游节，石风景名胜区和中信高尔夫海滨度假村被评为国家4A级旅游区。

　　——市场经济秩序和社会秩序明显好转。认真贯彻省委、省政府汕头现场办公会精神，大力整治市场经济秩序和社会秩序。制订用三年左右时间把汕头建设成为全国信用最好的城市之一的奋斗目标，在全省率先开展社会信用体系建设，开通全国首个地方政府社会信用网，在政府、公务员、市民、企业、中介机构等五个层面推进信用建设，出台公务员信用守则、市民信用公约、行政事业单位对外守信承诺，在全社会形成守信光荣、失信可耻的氛围。查处涉嫌偷骗税税额100万元以上企业141户，打掉偷骗税犯罪团伙7个，捣毁制假发票窝点10个；查处走私案件936宗，案值7．79亿元；查处涉假案件8089宗，捣毁制假窝点1461个；查处地方金融机构违法违纪案件124宗。市领导分工负责，组成工作班子，经过艰苦努力，妥善处置了一批经济遗留问题。“严打”斗争持续开展，社会治安综合治理成效显著。近两年刑事发案率持续下降，黑恶势力得到有力打击，群众反映强烈的盗窃机动车、“两抢”犯罪发案明显减少。

　　——经济体制改革继续深化。五年共有181家公有制企业实施不同形式的改革。民营企业参与国有企业改革实现新突破，汕头酿酒厂等被民营企业整体购并。一批民营企业改组为有限责任公司或股份有限公司。出台鼓励投资基础设施和公用设施政策，公路运输站场建设和经营向社会开放。公共财政基本框架初步建立。编制部门预算、国库集中支付制度试点逐步扩大，政府采购制度稳步实施。税收征管体制改革深入开展，市区实行税收属地征管。土地有形市场、有形建筑市场和产权交易市场初步形成，经营性土地使用权和政府投资工程实行公开招投标。市场办管分离工作全面完成。

　　——对内对外开放迈出新步伐。五年进出口总额208．34亿美元，远洋贸易在出口中的比重由1997年的41．3％提高到65．2％。去年民营企业出口4．4亿美元，占全市出口总额的28％。对外经济合作与交流进一步加强，五年引进外资项目697宗，实际利用外资22．2亿美元。伊士曼柯达、加德士石油等11家世界500强企业在汕投资。保税区建设稳步推进。海关、检验检疫等口岸查验单位积极支持地方建设。我市多次在国内城市举办商品展销会，提高“汕货”知名度。闽粤赣区域经济协作发展空间不断扩大。建筑企业在国内40多个城市承建工程，五年在外完成施工产值205．9亿元，占全市施工产值的51％。

　　——基础设施和市政建设进一步完善。五年全社会固定资产投资完成582．32亿元，其中投入基础设施和市政建设资金106．33亿元。石大桥、汕汾高速公路、珠池港区二期工程、龙珠水质净化厂等一批重点项目相继建成投入使用。新建万吨级以上泊位5个，新增港口吞吐能力500万吨。新建和改造公路635．6公里，公路密度每百平方公里达87．3公里。去年，邮政电信业务收入33．4亿元，电信装备技术水平和通信能力居全国中等城市先进行列。

　　城乡建设取得新成效。完成市、县（市）、镇土地利用总体规划编制。修编城市总体规划获国务院批复。汕头市行政区划调整工作基本完成。市政设施日臻完善，光华桥、解放桥、乌桥改造竣工通车。旧城区改造步伐加快，拆除危旧房屋52．8万平方米，新建楼房115万平方米。至去年，市区建成区面积99．3平方公里；城市绿化覆盖面积3214．3公顷，绿化覆盖率32．4％，人均公共绿地面积5．28平方米。市区五年新增公交线路22条，营运公共汽车平均每万人3．54标准辆。特区范围内基本实现垃圾无害化处理，城市环境综合整治定量考核成绩居全国前列。城市品位进一步提升，进入“国家卫生城市”、“国家环境保护模范城市”和“全国人防先进城市”行列。

　　——精神文明建设和社会各项事业取得新进展。以社会公德、职业道德、家庭美德为主要内容的公民道德建设主题活动广泛开展。文学艺术、社会科学、新闻出版、广播电视等事业不断发展，群众文化活动蓬勃开展。优秀传统文化得到弘扬，涌现一批精品力作。澄海被评为全国文化、广播电视、体育先进市。外事侨务工作上新台阶。落实农村侨房政策任务全面完成，落实市区城镇侨房使用权20万平方米，累计62．6万平方米。五年海外侨胞捐赠12．8亿元兴办公益事业，其中李嘉诚先生捐赠汕头大学8亿元。民政工作上新水平。社区建设稳步推进，龙湖区荣获“全国社区建设示范区”称号。去年全市殡改火化率达到省的要求。国防建设、民兵预备役和优抚安置工作得到加强，连续两次荣获“全国双拥模范城”称号。

　　科技进步取得新成果。去年实现高新技术产品产值120亿元。全市拥有国家级高新技术企业12家，省级高新技术企业75家，省级民营科技企业103家。汕头高新技术产业开发区继续发展。汕头软件园和金平、龙湖民营科技园等科技园区初具规模。一批国家和省市科技发展计划顺利实施。五年推广转化科技成果566项，转化率达到95％，新增产值75．27亿元。取得省内先进水平以上科技成果304项，获省级科技进步奖47项，取得专利授权4465件。成为“全国技术创新工程试点区域”和“国家电动汽车试验示范区”。南澳县被评为全国科技兴海示范基地。

　　教育、体育、卫生等各项事业快速发展。五年投入校舍建设资金12亿元，新建改建扩建校舍119．8万平方米。基础教育改革顺利开展，新增小学初中学位6万个，在校学生达到90．05万人。新增普通高中学位2．5万个。高考连年取得好成绩，去年录取率达78．4％。汕头职业技术学院建成招生。汕头大学办学水平进一步提高。全民健身运动广泛开展。竞技体育水平不断提高，我市运动员在1998年取得全国乒乓球锦标赛团体冠军，在第九届全运会上夺得金牌9枚。五年来，汕头籍运动员在国际比赛中夺得冠军30个。成功承办九运会跳水、乒乓球比赛项目。医疗卫生服务质量不断提高，非典型肺炎的控制取得阶段性胜利，重大疾病预防控制工作取得新成绩。五年筹资2．9亿元，新建扩建医疗用房14万平方米，医疗条件有较大改善。农村卫生工作有新进展。连续5年完成省下达的计划生育工作任务。气象、地震、保密、档案、地方志等工作取得新成绩。

　　——民主法制建设和政府自身建设进一步加强。坚持向市人大及其常委会报告工作、向市政协通报情况的制度，主动接受法律监督和民主监督。五年办理市人大代表议案、建议256件和市政协提案602件。顺利完成两届村委会换届选举和首届社区居委会民主选举，实现村（居）民自治。依法治市扎实推进。五年来，市政府提请市人大审议的法规草案15件，颁布政府规章50件，清理地方法规、政府规章和其他政策措施31718件。行政执法责任制全面推行，城市管理相对集中行使行政处罚权试点工作顺利进行。普法教育、法律服务工作得到加强。澄海被评为“全国‘三五’普法教育先进市”。民族宗教工作取得新成绩。审计、工商、物价、统计等工作积极为经济和社会服务。安全生产形势基本稳定。民间组织管理加强。严厉打击“法轮功”邪教组织成效显著。

　　政府职能和管理方式进一步转变。建立完善民主化、科学化、法制化决策机制，制订重大行政决策和规范性文件法律审查制度，重大决策基本程序得到规范。组建政务咨询专家库，聘请一批有研究、有实践的社会各界专家，作为市政府政务咨询委员，为市政府重大决策出谋献策。政务公开全面推行，行政规章、规范性文件和政府政策措施通过媒体向社会公布。政府部门和“窗口”单位普遍实行公开办事制度、收费公示制度和服务承诺制、首问责任制，设立投诉电话，接受社会监督，群众反映良好。顺利完成政府机构改革，市政府组成部门从38个减为30个，镇、街道从91个调整为71个，全市精减行政编制2088名。两轮行政审批制度改革取得重大进展，各类审批事项从改革前的1195项减为431项。取消、降低各类收费317项，年可减轻社会负担12．83亿元。整顿机关作风、机关作风评议、机关效能建设等活动扎实开展。设立企业投资管理服务中心，为企业提供方便快捷的服务。

　　——城乡人民生活不断改善。去年，市区居民人均可支配收入8740元，农村居民人均纯收入3984元。城乡居民储蓄存款余额470．5亿元，年均递增11．5％。城乡居民衣、食、住、行条件明显改善，家庭耐用消费品拥有量以及文化、教育、旅游、保健等消费支出逐年增加。坚持为民办好事实事，农村水改、住房解困、校舍修建、旧城和危桥改造、建设120急救中心、新增公交线路等一批重点项目按期完成。特别是大部分农村的群众饮用自来水问题基本得到解决，市区近2000户危房户和特困户喜迁新居，社会反响良好。

　　劳动就业和社会保障工作取得新成绩。出台促进就业再就业政策措施，去年城镇新增就业6万人，下岗职工实现再就业2．3万人。城镇登记失业率控制在2．8％以内。去年社保基金征缴额增长28．8％，10．3万名企业离退休人员的养老金100％按时足额发放。国有企业下岗职工基本生活保障、失业保险、城市居民最低生活保障三条保障线和国有集体企业特困职工基本生活优待继续实施。城镇职工基本医疗保险制度改革积极推进，全市参加基本医疗保险职工13．9万人。扶贫和农民减负工作取得新成绩。五年投入老区、山区、贫困地区建设资金2．6亿元。开展农民负担专项治理，去年减轻农民负担6584万元，全市农民负担控制在上一年人均纯收入的2．1％以内。

　　各位代表！过去五年取得的成绩，是省委、省政府和市委正确领导的结果，是市人大及其常委会和市政协监督支持的结果。这些成绩的取得，凝结着全市广大干部群众的智慧和艰辛的劳动。在此，我代表市人民政府，向全市人民，向驻汕部队、武警官兵、中央和省驻汕单位及社会各界人士，表示崇高的敬意！向所有关心、支持汕头现代化建设的港澳台同胞、海外侨胞和海内外友人，表示诚挚的感谢！

　　过去的五年，我市经济和社会发展遇到改革开放以来从未遇到过的困难，2001年国内生产总值出现负增长。面对困难，全市上下迎难而进，励精图治，一手抓整治、一手抓发展，以发展带整治、以整治促发展。经过不懈努力，国民经济呈现恢复性增长。汕头能有今天的局面，确实来之不易，五年的实践使我们深刻地体会到：必须全面贯彻落实“三个代表”重要思想，坚持把发展作为第一要务，才能真正维护和实现广大人民群众的根本利益；必须端正经济工作指导思想，坚持走正道、重信用、办实业、创品牌，才能真正把汕头的经济建设推上持续快速健康发展的轨道；必须大力推进体制创新，坚持建立和完善社会主义市场经济体制，才能真正为经济和社会发展注入不竭动力；必须切实转变政府职能，坚持把优化软环境作为生命线，才能真正提高经济综合竞争力；必须实施可持续发展战略，坚持把控制人口、节约资源、保护环境摆在十分重要的位置，才能真正实现经济和社会的协调发展；必须切实推进精神文明建设和民主法制建设，努力提高市民文明素质和城市文明程度，才能真正为经济和社会发展提供强有力的保障。

　　我们也清醒地看到，经济和社会发展中还存在不少困难和问题。主要是：产业结构性矛盾突出，产业层次偏低；工业基础还不牢固，经济发展后劲乏力；农村经济基础薄弱，农民收入增长缓慢；就业再就业压力大，城镇下岗失业人员增多，农村劳动力向非农产业转移难度大；财政刚性支出增多，压力较大；市商业银行等经济和社会发展的深层次问题还没有妥善解决。对此，我们必须高度重视，认真加以解决。今后五年的奋斗目标和政府工作安排

　　今后五年，是汕头经济和社会发展的关键时期。我们要认真贯彻落实胡锦涛同志视察广东和张德江同志视察汕头的重要讲话精神，按照市第八次党代会的要求，举全市之力，集全市之智，加快发展、率先发展、协调发展。

　　今后五年的政府工作，要认真贯彻市第八次党代会确定的工作指导思想，高举邓小平理论伟大旗帜，努力实践“三个代表”重要思想，围绕全面建设小康社会、率先基本实现社会主义现代化的总目标总任务，实施工业强市、城乡协调、外向带动、科教兴市和可持续发展五大战略，增创民营、侨乡、商贸、海洋四大优势，推进物质文明、政治文明和精神文明协调发展，把汕头建设成为现代产业协调发展、城乡经济整体推进的经济强市和区域性中心城市。今后五年，要全力提升工业化，加快城市化，推进现代化，构筑区域性信息、物流、文化“三个中心”；建设高新技术产业和先进工业、特色产业和传统工业、外贸出口加工和制造业、现代生态农业“四个基地”；营造依法办事、诚实守信、文明服务、清洁卫生、安全有序“五个环境”；抓好民营经济、外向型经济、园区经济、港口经济、商贸旅游经济、农村产业经济“六个重点”。经过五年的努力，达到下列目标：

　　——经济综合实力显著增强。到2007年，全市实现国内生产总值780亿元，年均增长10％左右，人均国内生产总值达到15800元；工业总产值1400亿元，年均增长11％左右；财政收入70亿元，年均增长11％左右；外贸出口总额25亿美元，年均增长10％左右。民营经济、园区经济和港口经济实现新突破，成为国民经济的重要支撑。纺织服装、工艺玩具生产基地进一步壮大，南区重化基地规划建设有实质性进展。电子信息、纺织服装、工艺玩具、化工塑料、食品医药、机械装备、印刷包装、音像材料八大支柱产业不断壮大，高新技术产业和先进工业体系初步形成。科技创新能力增强，信息化综合指数达到60％左右。物流、旅游、会展等现代服务业发展加快。对外开放进一步扩大，外向型经济跃上新台阶。农业产业化规模扩大，初步形成农业区域化合理布局、专业化分工协调的农村经济发展新格局。

　　——城市化水平大幅度提升。基本建成广东省域次中心城市，城市综合影响力和辐射力增强。城市公用设施、路网、港口、电力等一批新项目相继建设并完工，基础设施建设实现新跨越。市域交通网络化，城区建成快速环路，市区大部分地方可在半小时内上高速公路、1小时内到达机场和港口。市政建设有突破性进展，中心城区综合服务功能增强。城市化水平达到50％左右。生态环境明显改善，城市生活污水处理率达到70％以上，生活垃圾基本实现无害化处理，水环境污染得到有效控制。城市绿化水平持续提高，建成国家园林城市。

　　——社会主义市场经济体制更加完善。新的投融资体制基本建立，资本、技术、人才、劳动力等要素市场充分发育，有形建筑市场、产权交易市场、土地有形市场不断完善，市场配置资源的基础性作用进一步发挥。国有资产管理体制建立健全，国有企业改革任务全面完成。政府调节经济、监管市场、管理社会和公共服务的职能进一步增强。

　　——社会文明法治程度明显提高。政府行政行为规范高效，决策民主化、科学化、法制化程度显著提高。社会事业不断发展。九年义务教育高标准普及，高中阶段教育快速推进。逐步形成学习型社会。文化体育事业繁荣，文化产业规模化发展，文化大市和体育强市建设初具规模。精神文明建设进一步加强，公民道德水准显著提高，“汕头人精神”得到弘扬，建成全国和全省文明城市。民主法制建设进一步完善，社会治安稳定，人民群众安居乐业。

　　——人民生活实现宽裕小康。城乡居民收入不断增长，城镇居民可支配收入年均增长6％，农民收入明显提高。贫困村基本脱贫。城镇就业服务体系完善，下岗职工和新成长劳动力基本实现就业。社会保障水平明显提高，基本建立起覆盖城乡、比较完善的社会保障体系。医疗卫生和公共服务网络完善，人居环境明显改善，城乡人民生活富庶。

　　上述五年的发展目标，反映了人民群众的迫切愿望。实现人民的意愿，为人民谋福祉，各级政府责无旁贷、义不容辞。我们要始终坚持打基础、增后劲、快发展不动摇；坚持抓改革、求创新、保稳定不动摇；坚持为民办实事、送温暖、谋利益不动摇，一定要让人民群众在新一轮经济发展中得到更多的实惠！

　　2003年，是新一届政府的届首之年。做好今年的各项工作，对于实现今后五年政府工作目标至关重要。综合考虑诸多因素，今年全市经济和社会发展的主要预期目标是：国内生产总值增长8％左右，财政收入增长8％，工业总产值增长9％，全社会固定资产投资增长8％，外贸出口增长10％，社会消费品零售总额增长8％，城镇居民人均可支配收入增长3％，农村居民人均纯收入增长2％，城镇登记失业率控制在3．5％以内，人口自然增长率控制在8．5‰以内。

　　今后五年，政府工作要开拓新思路，谋求新发展，打开新局面。重点抓好以下十个方面：

　　（一）做强做大园区经济，大力发展先进工业

　　工业化是现代化的基础。要全面实施工业强市发展战略，以工业化带动城镇化，推进现代化。要牢固树立大办工业的思想，坚定不移地走新型工业化之路，着力引进工业项目，努力优化工业发展的环境。要以园区带动、产业带动、品牌带动，努力构建具有汕头特色、比较优势和较强竞争力的新型工业体系，使汕头工业从根本上变弱势为强势。

　　强化园区经济带动。园区经济是工业发展的重要载体，也是实现集约发展的重要途径。市和各区县都要高起点规划、高标准建设1至2个高新技术和先进工业园区，有条件的镇、街道也要规划建设工业园区。要结合行政区划调整，大气魄、大手笔、大动作对原有工业园区进行整合资源、扩大面积、形成规模，构筑以高新区、保税区、汕大科技园区和新规划的园区等一批市级工业园为龙头，金平和龙湖民营科技工业园、澄海岭海、濠江南山湾和潮阳、潮南工业园等区县工业园为骨干，一批镇、街道工业园为基础的上规模、上档次园区经济新格局。要把“筑巢引凤”和“引凤筑巢”结合起来，积极引进南区重化工业等大型特大型项目，促其配套自成园区或工业基地。工业园区不仅要做大，更要做优。要走可持续发展之路，依山傍水建设生态型工业园区。要把工业园区作为工业城来规划建设，集中开发，集中管理，集中服务，完善功能，提高效益，提升档次，促使工业项目向园区有效聚集。通过优化园区结构，构筑产业链，培育产业生态，推动企业集群发展，改变我市工业企业规模小、布局分散、质量效益不高的状况，使工业经济走新型工业化道路。

　　强化产业带动。积极推进产业结构调整和优化升级，在发展劳动密集型轻型工业和加工工业的同时，大力引进和发展高新技术和先进工业，重点扶持发展软件业、信息服务业、光机电一体化、生物工程、新材料、新能源等高新技术产业，改造提升传统产业，推动高新技术产业和传统产业协调发展。继续推进潮阳、潮南纺织服装、音像材料和澄海工艺玩具产业基地建设，重点扶持一批有发展潜力的大型企业集团，走规模扩张和质量提高之路。进一步完善扶持较大规模工业项目优惠措施，每年确定一批大项目作为工作重点，落实责任、加强服务、抓好协调、跟踪落实。今年，要抓好135项投资额1000万元以上的续建、新建工业项目建设，突出抓好其中33项投资额5000万元以上的项目；抓好16个重点技改项目和5个列入国债技改专项计划的重点项目建设。

　　强化品牌带动。扩大品牌效应，大力开展科技创新，广泛应用信息技术、高新技术和先进适用技术，不断提高核心技术和重要应用技术的创新能力，推动金刚玻璃等一批具有自主知识产权的先进技术和产品的开发运用，打响汕头品牌，提高自主知识产权经济在工业经济中的比重。引导和支持企业应用专利技术，做好质量体系认证、商标注册、专利申请。加快采用国际标准和国外先进标准组织生产，扶持、重奖创立名牌产品的企业，争创更多的国家级、省级品牌。

　　（二）全力招商引资，大力发展内源型和外源型经济

　　汕头经济外向度比较高，又蕴藏着巨大的民资民力，必须充分利用国际国内两种资源，让一切创造社会财富的源泉充分涌流迸发，掀起新一轮的投资兴业热潮。

　　加快发展民营经济，做大内源型经济。我市的民营经济正在蓄势待发，将迎来新一轮腾飞。我们要以更加开放的气魄，释放民资民力民智，鼓励民间资本投资兴业，促进民营经济大发展，建成民营经济大市。要创造宽松的社会环境，依法保护民营企业的合法财产和权益。营造良好的政策环境，引导鼓励民间资本进入竞争性和对外资开放领域，重点扶持科技型、外向型、就业型、农产品加工型民营企业。加强政府宏观指导，发挥商会和行业协会的作用，为民营企业提供社会化服务体系。引导民营企业规范运作，建立现代企业制度。引导有实力的民营企业设立市场化运作的信用担保机构，开辟民营企业融资新渠道。有条件的区县要兴办民营科技园区，引导民营企业提升产业层次。从今年起连续五年，市财政每年安排扶持中小企业专项资金3000万元，支持民营企业创新技术和开拓市场。

　　实施外向带动战略，做大外源型经济。要推动出口主体、出口方式、市场和产品的多元化，千方百计扩大外贸出口。提升加工贸易水平，加快一般贸易出口。优化出口商品结构，扩大机电产品等特别是高新技术产品出口。组织和引导企业走出去，开展境外投资和承包工程业务。大力发展远洋直接贸易，推动更多企业进入跨国公司全球供应链。要落实鼓励外贸出口的各项政策措施，加快出口退税进度，继续做好“免、抵、退”税工作。进一步放开企业进出口经营权，扶持更多的民营企业自营进出口业务，培育新的外贸增长点。要充分发挥港口城市优势，鼓励企业进口国内急需的原材料和设备，做大进口贸易。加强与海关等口岸查验单位的协调，营造方便快捷的通关环境。继续推进汕头与港澳台的经贸合作与交流，争取汕头与台湾南部港口海上直航工作有新进展。

　　多渠道全方位招商引资。充分发挥侨乡优势，积极推介汕头投资环境，实行以侨引侨，以侨引外，借侨兴市，大力吸引侨资、外资、内资。鼓励旅居海内外的汕头人回家乡投资参与建设，把“汕头人经济”变成“汕头经济”。建立“政府扶持、市场运作、效益挂钩”的投资促进机制，瞄准重点地区、重点项目、重点对象、重点领域，创新招商方式，通过专业招商、委托招商、网络招商、以商引商等，积极引进跨国公司、大财团和国内知名企业来汕投资。建立激励机制，重奖引资有功人员。建立优质高效的服务机制，以诚挚的感情和优质服务留住在办企业，做到爱商、亲商、富商、留商，促其增资扩产，影响和带动新的投资者。通过营造低成本、高效率、安全文明的投资环境，使汕头真正成为吸引外来投资的热土。

　　（三）高起点高标准规划，实现城乡协调发展

　　规划是城市的灵魂。要抓住行政区划调整、市区范围扩大的有利时机，加快城市化、城镇化、城乡一体化进程，努力形成城乡良性互动、产业协调发展的格局。

　　实施汕头城市总体规划。要找准未来城市发展定位，科学调整城乡布局，促进资源合理配置，扩大城市规模，完善城市功能，增强区域性中心城市的辐射带动能力。以全市概念性规划为指导，切实做好城镇化发展、产业布局、交通能源基础设施建设、土地利用、园林绿化与环境保护、海洋功能区划等专项规划的修编。各区县要在市总体规划的指导下，做好分区规划，形成“大汕头”布局。

　　构筑城乡发展新格局。未来汕头城市发展要以金平、龙湖、濠江为城市中心，澄海、潮阳、潮南城区为副中心，南澳为前沿，中心镇和一批新兴的城镇为骨干，构筑主、副中心城区和重点城镇布局合理、产业协调、发展有序的市域城镇体系。各区县要按照产业功能定位，发挥优势，突出特色，大力发展壮大区县域经济。金平区、龙湖区要大力发挥城市中心区位优势，发展商贸、物流、会展、金融、房地产、信息、旅游等第三产业，重点发展电子信息、机械装备、印刷包装、医药等先进工业，建设汕头行政、经济和文化中心。濠江区要借助港口优势，积极引进大型特大型工业项目，发展能源、重化、临港工业和先进工业，规划建设港口物流业，大力发展旅游业和建筑业。澄海区要增创“中国玩具礼品城”品牌优势，建设国际知名的玩具礼品生产基地和商品集散地，发展纺织服装、食品加工、化工建材等特色产业和先进工业，大力发展特色农业和生态旅游业。潮阳区、潮南区要建设成为国内外知名的纺织服装、音像制品、高新技术和先进工业生产基地，发展精细化工、文具用品等产业，成为特色轻工产品集散地，大力发展港口经济和“三高”创汇农业。南澳县要大力发展旅游业、海洋产业、环保产业和生态农业，建设生态旅游区和国家中心渔港。

　　（四）实施城市化发展战略，适度超前推进基础设施建设

　　基础设施是城市的形象，是城市综合竞争力的关键。要围绕建设经济强市，适度超前规划建设一批事关汕头长远发展的重大基础设施，做到规划一批、建设一批、储备一批，拉动经济持续快速发展。

　　加快交通能源基础设施建设。建设以港口为龙头，城乡道路为基础，高速公路和铁路为骨架，海港、空港为枢纽，客货站场为配套，通信为平台，布局合理、设施先进、功能完善、安全高效的海陆空交通运输网络，把汕头建成区域性物流、客流中心。汕头港要充分发挥全国沿海主枢纽港功能，增强港口辐射力。进一步完善珠池港区配套建设，重点建设广澳港区。加快广澳港区一期工程5万吨级泊位建设，规划论证广澳港区二期工程10万吨级泊位项目，积极推进广澳港区港口公共设施建设，抓紧组织实施5万吨级航道工程及西防波堤建设，尽快筹建大型集装箱、成品油、原油、石化等专用码头泊位。加快发展海运业，增辟新航线，进一步发挥港口效益。完善路网建设，加快汕头至揭阳高速公路、澄海金鸿公路、潮阳和潮南路网改造、公路主枢纽站场等工程项目建设，积极做好潮阳至揭阳高速公路和汕漳、汕深、广澳港疏港铁路等项目的规划及前期工作。抓紧实施国道324线潮南段和外砂、莲阳、东里大桥扩建。加快电力建设，动工兴建华能汕头电厂二期工程60万千瓦机组，跟踪落实建设华能南澳风电场前期工程、南澳丹南和英华威风电项目，做好华能汕头第二电厂2×90万千瓦机组项目的前期工作，使能源建设在今后几年有一个新飞跃。

　　推进市政设施建设。着手规划建设市区快速环路，配套完善城区道路网。要做好一批重点市政设施项目的上马准备工作，按先后缓急分期建设。力争尽快完成金凤大桥－西港高架桥、石大桥北引道等重点建设项目。续建黄山路、韩江路等城市道路。开工建设海滨路西延工程、金砂东路东延工程、明珠大桥等市政项目。加快旧城改造步伐，重点推进小公园历史建筑风貌保护区的建设。建立完善以公共交通为主、多种方式有机结合的综合交通体系。推进中心城区环保基础设施工程的建设，今年要完成雷打石生活垃圾填埋场第一期工程，启动南区等垃圾处理设施建设。规划建设大型生活垃圾焚烧厂，实行垃圾安全化和无害化处置。完善龙珠水质净化厂截污工程建设，规划建设西区和澄海、濠江、潮阳、潮南污水处理厂及南澳污水处理工程。全面实施“青山、碧水、蓝天、绿地”工程，加快城市出入口绿化和江、河、海岸景观建设，营造涵养林、生态林，建设城市“绿环”，保护牛田洋、苏埃湾大型湿地。巩固“创卫”、“创优”、“创模”成果，创建国家园林城市、国家生态城市。

　　加强水务设施建设。按百年一遇的防洪潮标准全面加固建设汕头大围，加紧完成江海堤围、水库、水闸、电排等除险加固工程，提高整体防灾抗灾能力。建设第二过海水管。规划整治练江、濠江、潮阳护城河、澄海环城河、中心城区西港内河、龙湖沟、新河沟、黄厝围沟、潮南峡山溪，加快河流治理步伐。

　　（五）大力发展农村经济，实现农业增效农民增收

　　建设经济强市，必须把“三农”问题摆在突出位置，更多地重视农村，关注农业，关心农民。

　　调整优化农业经济结构。大力发展蔬菜、水产、畜牧等主导产业，建设韩、榕、练三江平原地带粮菜轮作农业区、城郊“菜篮子”农业区、山区和沿山地带果林生态农业区、江河水网地带立体养殖生态农业区、南澳海洋产业区等五大特色产业区。突出发展特色农业、订单农业和创汇农业，拓展“品牌农业”，培育一批名优特农产品，建设一批“一镇一业”、“一村一品”生产基地。大力发展生态农业，调整和优化农业生态结构，实现农业生产和生态良性循环。制订完善地方农产品标准，加快农产品商标注册和国际质量体系、有机食品、绿色食品等认证步伐，逐步实现农产品生产标准化。实施“无公害食品行动计划”，继续建设澄海万亩无公害蔬菜基地以及水产养殖无公害示范基地，全面提高农产品质量安全水平。推进科技兴农，建设农业现代化示范区。切实保护耕地，认真落实土地用途管制和占补平衡制度。加快土地使用权流转制度改革，做好弃耕丢荒土地的复耕工作，鼓励引导土地使用权向生产能手集中，扩大经营规模。合理开发利用海洋资源，积极发展近海养殖业和远洋捕捞业，抓好水产养殖和水产品加工出口基地建设，加快海洋产业化进程。支持云澳、海门渔港建成国家中心渔港。

　　提高农业产业化水平。大力推进农业产业化，依托产业带动，发展效益农业。培育农业龙头企业经营体系，发展一批以市场、加工、科技带动型为主的企业集团；建立和完善“公司＋基地＋农户”模式，把分散的农户与市场联结起来，推动农业商品化、专业化、产业化。创新生产经营体制，通过财政资金和政策扶持，重点培育科技型龙头企业、农产品加工型龙头企业。构建农产品加工体系，提高农产品加工率、附加值和综合经济效益。完善农产品流通体系，发展各类运销中介组织，重点提升农副产品、水果、水产批发市场的档次。

　　实施农村税费改革。严格执行国家和省农村税费政策，切实减轻农民负担。规范农村税费管理，保障农村基层政权运转和农村义务教育的落实。积极探索农业税收征管体制改革，进一步规范农业税收征管行为。

　　加大扶贫开发力度。加强农村政权建设，选好善开拓、懂管理、会经营的能人，找准经济发展的路子，大力发展农村集体经济，切实解决集体经济零分配问题。认真落实“十五”扶贫开发计划，重点扶持132个集体经济年纯收入3万元以下的贫困村，实行市直机关扶贫、经济发达的镇、村帮贫等形式，争取用三年时间实现基本脱贫；确保扶持政策和配套资金到位，帮助山区、老区和贫困地区人均年收入1500元以下的群众改善生产和生活条件。加快农村公路建设，从今年起连续五年，市政府每年安排1000万元支持农村公路建设，实现村村通水泥路。继续做好农村水改工作，实现村村通自来水。要逐步从“输血型”扶贫向“造血型”扶贫转变，推进科教文化扶贫，帮助贫困村增强自我发展能力。

　　（六）扩大有效需求，实施消费拉动

　　扩大内需，实施消费拉动是经济发展的积极有效措施。必须增创“百载商埠”新优势，整合商贸服务业资源，构建适应区域性中心城市特点、辐射功能强的商贸服务业体系，满足日益增长的社会消费需求。

　　努力扩大消费需求。采取政府推动、商会行业协会搭台、企业唱戏等形式，繁荣本地市场，开拓农村市场，进军内地市场，进一步刺激消费。办好商业步行街和现代连锁商业，改造肉菜市场，扩大和规范连锁生鲜超市，改善消费环境。合理引导消费，鼓励住宅、汽车等大宗信贷消费，培育信息服务、教育、文化、保健、休闲等新消费热点。规范垄断行业和服务业的价格行为，全面推行明码标价和收费公示制。培育会计、审计、法律、房地产等中介服务，发展社区服务业。

　　加快发展现代服务业。充分发挥港口优势和区位优势，依托海港、空港、铁路等交通枢纽和保税区，加快区域性现代物流中心建设步伐。发展现代物流配送业，构建工商企业物流平台、准时运输系统、社区配送系统。大力发展会展经济，发挥中国进口商品汕头博览中心的作用，加强与各国会展协会的联系，每年组织若干次大型全国性会展，探索举办国际性行业会展。今年要办好首届汕头国际纺织服装交易会、国际食品博览会、澄海国际玩具工艺品博览会等大型展会。加快发展旅游业，办好每两年一届的汕头市旅游节，开发具有鲜明地方文化特色的旅游景区和旅游商品，建设“中国美食之都”，打造汕头特色旅游品牌。力争南澳县进入国家4A级旅游区行列。

　　（七）加大经济体制改革力度，为经济社会发展提供动力

　　改革是发展的强大动力。要大胆探索，敢为人先，突破阻碍生产力发展的体制性障碍，创新体制，健全市场体系，激活市场主体，推动经济发展。

　　大力推进投融资体制改革。实施投资拉动，需要大量的资金投入。要创新思路，确立新理念，坚持“有所为、有所不为”的方针，按照“政府主导、市场运作、社会参与”的原则，大力培育社会投资主体，大胆利用社会各种资金参与经营性和公共基础设施项目的建设，为我市重大基础设施建设多渠道筹措资金。凡是能推向社会和市场的投资项目，都推向社会，实行市场运作。凡是该由政府投资的项目，实施积极的财政政策，适当举债建设，发挥政府投资的杠杆作用。积极采取多种融资方法，利用银行信贷、世行贷款、出让经营权、BOT、TOT等融资方式参与污水处理厂、垃圾处理厂、公路、港口等建设。对道路、桥梁的建设，要尽量少采用收费偿还投资的运营方式，以利改善投资环境。所有基础设施建设项目，都必须进行招投标，加强监理，做到高起点规划，高质量建设，高效能管理，争取获得最大的经济效益和社会效益。推进政府资源市场化、资本运作专业化。大力推行“经营城市”模式，实行旅游资源、土地资源、海洋资源、城市公共资源和地下管网资源等政府可支配资源的有偿使用，加快公用服务产业化，使城市在经营中增值和发展。

　　加快国有企业改革步伐。坚持“先易后难、分类处置、整体推进”的原则，加大国有企业改革力度，盘活国有企业存量资产。以产权制度改革为主线，多形式加快国有企业的改制改组改造。积极鼓励民资外资参与国有企业改革，在竞争性领域和行业实现“国退民进”。采取适度运用借贷资金提前支付改革成本等有效手段，做好安置国企职工工作，加快改革进度。大胆实施民事破产，建立劣势企业退出机制。今年，抓紧对列入改革计划的227户市直国有企业、54家企业化管理的事业单位实施改革。本届政府将在任期内完成国有企业的改革任务。

　　深化国有资产管理体制改革。成立市国有资产管理委员会和国有资产管理部门，统一行使国有资产管理职能，形成名副其实的管资产管人管事相结合、责权利相统一的国有资产管理体制。组建若干家国有资产经营公司，探索建立有效的国有资产经营机制和运作方式，实现国有资产合理配置及保值增值。

　　继续推进财税改革。全面推进依法治税，培育税源，加强征管。推进税收信息化建设，实行税银库企一体化。围绕构建公共财政框架的目标，全面推行公共财政支出管理改革，保持财政平稳运行。扩大政府采购的范围和规模，完善政府采购制度。全面实行市直行政事业单位编制部门预算，逐步扩大国库集中支付试点范围，年底前全面实行国库集中支付。按照财权、事权相统一原则，探索改革理顺市、区财政管理体制，多方面调动积极性。提高公务员福利待遇，今年7月1日起，对市直预算单位实行统一公务员岗位津贴。进一步强化审计监督力度，加强对重点领域、重点部门、重点资金的审计监督。

　　（八）加强文化建设，协调发展社会各项事业

　　文化建设是社会主义现代化建设事业的重要组成部分。必须依靠科技、教育和人才的智力支撑，加快发展社会各项事业，不断增创新优势，实现建设经济强市的奋斗目标。

　　实施科教兴市战略，推进人才资源开发。继续抓好国家技术创新工程试点，建立健全企业技术创新机制，推进科技创新和高新技术产业化。努力创建“国家火炬计划光机电产业基地”。组织和引导企业加强与清华大学、北京大学、中科院等科研院校的合作，推进产学研联合，促进科技成果转化为现实生产力。开展科普工作，提高全民科学文化素质。优先发展教育。全面实施素质教育，巩固提高九年义务教育，金平、龙湖、濠江区和澄海、潮阳、潮南城区以及南澳县城要基本普及高中阶段教育。积极发展职业技术教育和高等教育，大力推进教育信息化。整合教育资源，调整学校布局。落实“以县为主”的基础教育管理体制。增加教育投入，加强学校经费收取和使用的监管。继续支持办好汕头大学，充分发挥汕头大学作为人才、科研基地的优势。要切实做好人才培养、引进、使用和管理工作，建立人才激励机制，建设人才高地。充分发挥“京粤博士后汕头科技开发基地”、“博士后创业园”、“留学回国人员创业园”的作用。深化人事制度改革，推进整体性人才资源开发，营造使人才引得进、用得上、留得住的良好环境，把优秀人才聚集到汕头现代化建设事业中来。

　　加强文化建设，发展社会各项事业。以建设文化大市为目标，抓紧制订五年文化发展战略。学习宣传《公民道德建设实施纲要》，深入开展“汕头人精神”大讨论，大力倡导与时俱进、昂扬向上的时代精神。积极开展群众性文化活动，丰富群众文化生活。弘扬传统优秀文化，推出更多的文化精品。大力发展文学艺术、社会科学、新闻出版、广播电视等文化事业。加强城乡文化基础设施建设，深化文化管理体制改革。抓紧制订潮汕人文特色的文化产业发展规划，加快文化产业发展。推进卫生体育事业。建立健全突发公共卫生事件应急反应机制和疾病预防控制体系，切实保护人民群众的身体健康和生命安全。继续深化城乡医疗卫生体制改革，完善医疗机构管理和运行机制，推进社区卫生服务工作，改善农村医疗卫生条件。完善药品集中招标采购制度，规范医疗收费和药品价格行为。进一步加强人口与计划生育工作，落实目标管理责任制，建立调控有力、管理有效、政策法规完备的计划生育工作机制和保障机制，实现稳定的低生育水平。实施全民健身计划，提高竞技体育训练水平。培育体育市场，发展体育产业。加快中国乒乓球队汕头训练基地建设，积极做好承办第二十届世界华人篮球邀请赛的准备工作。加强国防教育，推进国防建设，抓好民兵预备役工作。做好拥军优属、优抚安置工作，大力支持驻汕人民解放军和武警部队建设，深入开展创建双拥模范城活动。继续推行殡葬改革，倡导移风易俗。规范社团登记工作，加强民间组织管理。加快发展妇女儿童事业，维护妇女儿童合法权益。进一步做好宗教、对台、外事、侨务、档案、保密、地方志等工作。

　　千方百计扩大就业，大力发展社会保障事业。就业是民生之本。要认真落实就业和再就业的各项政策措施，致力营造良好的就业环境。拓宽就业渠道，着力新增工业、社区服务、商贸、餐饮、旅游、园林绿化、医疗保健、城市保洁、保安等就业岗位。鼓励劳动者实行非全日制等弹性就业。开拓市外、境外劳务市场，做好劳务输出工作。依法保障劳动者合法权益。加大职业技能培训力度，构建以公益性为主、社区和民办为辅的职业介绍服务网络。巩固和扩大社会保险覆盖面，不断完善养老、失业、工伤、生育和医疗保险制度。加强社会保险费征收和监管，推进社保费市级统筹和全市社保费由地税部门全责征收。确保国有企业下岗职工基本生活，确保离退休人员养老金按时足额发放。全面推进国有企业下岗职工基本生活保障向失业保险并轨。逐步推行企业离退休人员社会化管理。加强救灾救济工作，规范和完善城市居民最低生活保障制度，将所有符合条件的贫困居民纳入低保范围。积极开展经常性社会捐助活动。力争经过三年的努力，基本建立起覆盖城乡、比较完善的社会保障体系，使社会保障达到较高水平。重视老龄人口问题，实施老年事业发展“十五”纲要。关心残疾人事业，推进残疾人康复和就业。

　　（九）加强民主法制建设，维护社会稳定

　　加强社会主义民主法制建设，是社会主义政治文明的重要内容。必须继续贯彻依法治市方针，切实提高依法行政水平，全力维护社会稳定。

　　推进民主法制建设。坚持市政府向市人大及其常委会报告工作、向市人民政协通报情况的制度，自觉接受人大的法律监督和政协的民主监督，接受舆论和人民群众的监督。认真办好人大代表议案、建议和政协委员提案。发挥民主党派、工商联、无党派人士参政议政作用和工会、共青团、妇联组织的桥梁纽带作用。加强政府立法工作，提高立法质量，完善行政规章。加强行政执法队伍建设，规范行政执法行为，强化行政执法监督。增强公职人员的法律素质，提高依法行政水平。深化全民普法教育，增强法制观念。进一步健全行政复议和法律援助机制。扩大基层民主，保证人民群众依法充分行使民主选举、民主决策、民主管理和民主监督权利，完善村民自治和城市居民自治，全面推进社区建设。坚持和完善以职工代表大会为基本形式的企事业民主管理制度，保障职工的合法权益。健全社情民意反映制度，做好群众来信来访工作，妥善化解社会矛盾。

　　维护市场经济秩序。继续抓好社会信用体系建设，完善信用网络建设，加强信用立法，推进信用服务市场化运作。进一步改善金融环境，严厉打击逃废债务行为，建立金融安全区。加大对市商业银行的处置力度，积极妥善解决各种经济遗留问题。深入开展整治市场经济秩序，坚决打击走私贩私、制假售假、逃税骗税、逃废债务等违法犯罪活动。强化各级安全生产责任制，开展安全生产专项整治，遏制重大安全生产事故发生。

　　强化社会治安综合治理。坚持“打防结合，预防为主”方针，坚决打击侵害人民群众生命财产安全的“两抢”、盗窃机动车等多发性犯罪活动，严厉打击暴力性犯罪、涉恶涉黑犯罪，扫除“黄赌毒”等社会丑恶现象，净化社会治安环境。巩固严打整治斗争成果，构建长效治安防控体系。加强国家安全工作，坚决打击敌对势力和“法轮功”邪教组织的破坏活动。

　　（十）强化政府自身建设，优化发展软环境

　　软环境是发展的生命线。各级政府及公务员必须牢固树立一切为经济发展服务、一切为改善投资环境服务的思想，转变观念、转变职能、转变作风，建设廉洁、勤政、务实、高效的政府。

　　切实转变政府职能。深化行政管理体制改革，进一步加强政府提供公共产品、搞好公共服务、创造公平环境的职能。开展新一轮行政审批制度改革，继续削减审批事项，创新审批方式，简化审批程序。继续推进政企分开、政事分开、政社分开，把社会服务性职能交给中介机构，促进中介机构发展。改进行政管理方式，运用法律手段调节、管理经济和社会事务，提高政府依法行政能力。尽快探索建立适应中心城市发展特点的政府分级管理体制，明晰管理权限，理顺条块和层级之间的关系。积极推进城市行政执法试点工作。

　　继续推进政务公开。发挥政务咨询专家在政府重大决策中的参谋作用。严格重大决策的规则和程序，坚持咨询、论证、听证、法律审查制度，规范政府决策行为，提高决策的民主化、科学化、法制化水平。强化决策执行监督，确保政令畅通。加快推进政务信息化，完善政府公众网。加强效能建设，政府机关和对企业、群众的直接服务单位，全面实行政务公开，为群众提供公开、高效的政府信息服务。搭建全市行政服务平台，实现企业投资、生产经营所需审批、服务事项“一站式”完成。建立健全政府部门行政效能评估考核体系，全面提高政府各部门的办事效率。

　　加强政风建设和廉政建设。各级政府和公务员要进一步密切与人民群众的血肉联系，深入群众、深入基层，求真务实，反对官僚主义、形式主义，反对文山会海。始终牢记“两个务必”，勤政廉政，干净干事，做到权为民所用，情为民所系，利为民所谋，当好人民公仆。健全廉政机制，强化对行政权力的监督。切实纠正部门和行业不正之风，进一步从源头上预防和治理腐败。

　　坚持执政为民。在大力发展经济的同时，要着力解决好人民群众反映强烈的饮水难、住房难、就医难、入学难、行路难、就业难等“六难”问题，以实际行动赢得民心。今年要突出抓紧做好为民办好事实事的8个重点项目：解决部分群众住房难；解决中心城区商品房确权发证遗留问题；新建、改建、扩建一批中小学校舍，缓解中小学入学难；解决农村部分群众饮用自来水难；建设汕樟北路、黄河路接通泰山路等市政工程，进一步改善市区交通和居民生活环境；启动龙湖沟引韩冲污工程；启动中山公园改造工程；全面启动“社区老年福利服务星光计划”。上述8个重点项目，一定要精心组织，落实资金，加强督查，确保如期完成。

　　各位代表！我们欣喜地看到，汕头的经济和社会发展已经走出低谷，正以崭新的姿态站在新的起跑线上。展望未来，我们满怀信心。让我们团结在以胡锦涛同志为总书记的党中央周围，在市委的坚强领导下，高举邓小平理论伟大旗帜，全面贯彻“三个代表”重要思想，与时俱进，开拓创新，真抓实干，为把汕头建设成为经济强市和区域性中心城市而努力奋斗！
